# Evolutionary Diversification of *SPANX-N* Sperm Protein Gene Structure and Expression

**DOI:** 10.1371/journal.pone.0000359

**Published:** 2007-04-04

**Authors:** Natalay Kouprina, Vladimir N. Noskov, Adam Pavlicek, N. Keith Collins, Pamela D. Schoppee Bortz, Chris Ottolenghi, Dmitri Loukinov, Paul Goldsmith, John I. Risinger, Jung-Hyun Kim, V. Anne Westbrook, Gregory Solomon, Hanna Sounders, John C. Herr, Jerzy Jurka, Victor Lobanenkov, David Schlessinger, Vladimir Larionov

**Affiliations:** 1 Laboratory of Molecular Pharmacology, National Cancer Institute (NCI), National Institutes of Health, Bethesda, Maryland, United States of America; 2 Genetic Information Research Institute, Mountain View, California, United States of America; 3 Department of Cell Biology, University of Virginia Health Systems, Charlottesville, Virginia, United States of America; 4 Laboratory of Genetics, National Institute on Aging (NIA), National Institutes of Health, Baltimore, Maryland, United States of America; 5 Laboratory of Immunology, National Institute of Allergy and Infectious Diseases, Bethesda, Maryland, United States of America; 6 Department of Biochemistry and Molecular Genetics, University of Virginia, Charlottesville, Virginia, United States of America; 7 Laboratory of Molecular Carcinogenesis, National Institute of Environmental Health Sciences (NIEHS), National Institutes of Health, Research Triangle Park, North Carolina, United States of America; University of Texas Arlington, United States of America

## Abstract

The sperm protein associated with nucleus in the X chromosome (*SPANX*) genes cluster at Xq27 in two subfamilies, *SPANX-A/D* and *SPANX-N*. *SPANX-A/D* is specific for hominoids and is fairly well characterized. The *SPANX-N* gave rise to *SPANX-A/D* in the hominoid lineage ∼7 MYA. Given the proposed role of *SPANX* genes in spermatogenesis, we have extended studies to *SPANX-N* gene evolution, variation, regulation of expression, and intra-sperm localization. By immunofluorescence analysis, SPANX-N proteins are localized in post-meiotic spermatids exclusively, like SPANX-A/D. But in contrast to SPANX-A/D, SPANX-N are found in all ejaculated spermatozoa rather than only in a subpopulation, are localized in the acrosome rather than in the nuclear envelope, and are expressed at a low level in several nongametogenic adult tissues as well as many cancers. Presence of a binding site for CTCF and its testis-specific paralogue BORIS in the *SPANX* promoters suggests, by analogy to *MAGE-A1* and *NY-ESO-1*, that their activation in spermatogenesis is mediated by the programmed replacement of CTCF by BORIS. Based on the relative density of CpG, the more extended expression of *SPANX-N* compared to *SPANX-A/D* in nongametogenic tissues is likely attributed to differences in promoter methylation. Our findings suggest that the recent duplication of *SPANX* genes in hominoids was accompanied by different localization of SPANX-N proteins in post-meiotic sperm and additional expression in several nongonadal tissues. This suggests a corresponding functional diversification of *SPANX* gene families in hominoids. SPANX proteins thus provide unique targets to investigate their roles in the function of spermatozoa, selected malignancies, and for SPANX-N, in other tissues as well.

## Introduction

Mammalian spermatogenesis is a complex hormone-dependent developmental program in which a myriad of events ensure proper development of germ cells at the right time. The genes expressed during spermatogenesis comprise diploid and haploid expressed groups [1]. Many of the haploid, post-meiotically expressed genes have been mapped to autosomal chromosomes, but the sperm protein associated with nucleus in the X chromosome (*SPANX*) gene family is one of the few mapped to the X chromosome [2]–[4].

The Xq27 *SPANX* multigene family includes two subfamilies, *SPANX-A/D* and *SPANX-N*. *SPANX-A/D* has five members, *SPANX-A1*, -*A2*, -*B*, -*C*, and –*D*, which are extensively characterized. Each has two exons separated by a ∼650 bp intron containing a retroviral long terminal repeat (LTR) [2]–[4]. Further classification of *SPANX-A/D* genes is based on the presence of diagnostic amino acid substitutions, with one group (97 amino acid proteins) containing *SPANX-A1*, -*A2*, -*C*, and –*D*, and the other comprised of the *SPANX-B* gene (103 amino acid protein) that varies to up to as many as a dozen copies [5].

The SPANX-A/D proteins were first detected in the nuclear envelope of early round spermatids in the Golgi phase of acrosomal biogenesis. As nuclear condensation and elongation proceed, SPANX-A/D proteins migrate as a distinct post-acrosomal domain of the nuclear envelope towards the base of the nucleus. In the mature spermatids, SPANX-A/D proteins then associate with the redundant nuclear envelope within the residual cytoplasm. The SPANX-A/D domain of the nuclear envelope is thus caudal to the acrosome and reorganized as acrosome biogenesis progresses, ultimately constricting into the redundant nuclear envelope. Interestingly, only 50% of ejaculated spermatozoa showed staining of the nuclear craters and cytoplasmic droplet, corresponding to the redundant nuclear envelope with SPANX-A/D specific Abs [4], [6]. The localization of SPANX-A/D to a subpopulation of spermatids and spermatozoa suggests the precise temporal and spatial distribution of SPANX-A/D proteins in post-meiotic spermatid nuclei. In accordance with a special role of SPANX-A/D proteins in spermatogenesis, expression of these genes was not detected in nongametogenic adult tissues [4], [6]. They were, however, found expressed in various malignancies [7]–[12], making them conceivable candidates for cancer immunotherapy.

Ironically, the *SPANX-N* genes were discovered later than *SPANX-A/D* but prove to include their ancestral precursor. Presumed to be present in all mammals, they gave rise to the *SPANX-A/D* subfamily in the hominoid lineage ∼7 MYA [13] and consist of five members. Four *SPANX-N* genes (-*N1*, -*N2*, -*N3*, and -*N4*) are mapped ∼1.3 Mb away from the *SPANX-A/D* gene cluster. Each of these genes has ∼8 kb intron containing the ERV sequence flanked by two long terminal repeats (LTR). The fifth member, *SPANX-N5*, is located on the short arm of the X chromosome at Xp11. SPANX-N proteins share 60–80% identity with each other and 40–50% similarity with the sequences of SPANX-A/D proteins; all encode unfolded small proteins with a similar organization of coding and noncoding regions [13], though two SPANX-N proteins, SPANX-N1 and SPANX-N2, have a frameshift mutation in exon 2 that suggests they may not be functional.

Here we describe an analysis of the distribution of *SPANX-N* gene expression, protein localization within spermatozoa, and some features of polymorphism and evolution. Despite the structural similarities of *SPANX-A/D* and *SPANX-N* subfamilies, we find that they differ greatly in expression pattern and localization site in spermatozoa. Taken together, these results suggest that duplication of *SPANX* genes in primates was accompanied by diversification of gene function.

## Methods

### Tissues and cell lines

SKOV3 and ten melanoma cell lines 537MEL, 938MEL, 1363MEL, 501AMEL, 526MEL, 553BMEL, 624MEL, 836MEL, SKMEL28 and 888MEL were all established at the Surgery Branch of the National Cancer Institute, NIH (kindly provided by Steven Rosenberg). Melanoma cell line VMM150 was derived from a tumor digest obtained from a patient at the University of Virginia [14]. NCI-60 cancer cell lines that included six types of cancer (8 endometrial, 7 colorectal, 7 ovarian, 4 melanoma, 12 breast, and 5 prostate) were from the National Cancer Institute, NIH. Human normal tissues (prostate, placenta, proximal and distal colon, lung, cervix, uterus, stomach, testis, brain, liver, skeletal muscle, spleen, heart, lymphoma, lymph node, and kidney) were from Clontech Laboratories, Inc. (Mountain View, CA, USA); normal/tumor RNA pairs (ovary, prostate, uterus, breast, cervix, testis, lung, thyroid, colon and stomach) were from Ambion, Inc. (Austin, TX, USA). Primary tumors (ovarian and uterine) were kindly provided by Larry Maxwell, CCR, NCI, NIH). Tissues were obtained with Institutional Review Board-approved informed consent, and this study was approved by the NCI Institutional Review Board.

### Analysis of normal and cancer tissues by RT-PCR

Total RNA from normal adult human tissues, normal/tumor tissues pairs, NCI-60 cancer cell lines, melanoma cell lines and primary tumors was used for screening *SPANX-N* expression with the primers described in Table S1. cDNA was made from 1 µg of total RNA using the Superscript first strand system kit (Invitrogen, Carlsbad, CA, USA) and primed with oligo dT per their standard protocol. Human beta-actin primers (BD Biosciences Clontech, Mountain View, CA, USA) were used as positive controls. RT-PCR was performed using 1 µl of cDNA in a 50 µl reaction volume. Standard reaction conditions were 94°C 5 min, (94°C 1 min, 55°C 1 min, 72°C 1 min×35 cycles), 72°C 7 min, 4°C hold). To evaluate abundance of *SPANX-N* transcripts in nongametogenic tissues, a set of dilutions of the testis cDNA was done. The same intensity of bands in nongametogenic tissues was obtained when the testis cDNA was diluted 50–100 times. Before sequencing, PCR products were cloned into a TA vector (Invitrogen, Carlsbad, CA, usa). Database analysis was performed using versions of the BLAST program appropriate for different types of sequence comparisons: BLASTN for nucleotide sequences, BLASTP for protein sequences.

### Generation of peptide specific antibodies

Three synthetic peptides representing SPANX-N (EQPTSSTNGEKRKSPCESNN; positions 2–21), SPANX-B (ANEANEANKTMP; positions 21–32), and SPANX-C (SNEVNETMP; positions 18–26) were conjugated to Keyhole Limpet Hemacyanin and used as immunogens in rabbits according to an established protocol [15]. The resulting antisera (EQPT, ANEA, and SNEV) were affinity–purified over columns of peptide conjugated to Affigel 15 (Bio-Rad, Hercules, CA, USA) and concentrated in stirred cells with YM30 membranes (Millipore, Billerica, MA, USA). The concentrates were then subjected to gel filtration chromatography using 2.6×60 cm^2^ Superdex 200 columns (GE Healthcare, Piscataway, NJ, USA), and the monomeric IgG fractions were pooled and concentrated. The protein concentrations were determined using the Bradford assay (Bio-Rad, Hercules, CA, USA).

### Production of recombinant SPANX proteins and test for antibody specificity

For the production of SPANX proteins in *E. coli* cells, full-size ORFs of *SPANX-N1* (216 bp), *SPANX-N2* (540 bp), *SPANX-N3* (423 bp), *SPANX-N4* (297 bp) and *SPANX-N5* (216 bp) as well as *SPANX-B* (309 bp), and *SPANX-C* (291 bp) were generated by RT-PCR from RNA samples using the primers described in Table 1S and cloned into the *Bam*HI site of the pMAL-p2X expression vector (New England BioLabs Inc., Beverly, MA, USA) to produce a fusion maltose-binding protein (MBP). The recombinant fusion proteins were purified by affinity chromatography using a column with MBP. Expression of these fusion SPANX proteins was performed in TB1 bacterial cells. To produce non-fusion proteins, ORFs of all five SPANX-N genes were also cloned as *Bam*HI fragments into the pET-11d vector (New England Biolabs Inc., Beverly, MA, USA). Expression of these full-size SPANX proteins was performed in Bl21 cells containing an integrated copy of the T7 RNA polymerase gene. For one-dimensional SDS-PAGE, electrophoresis was performed on 4–20% Tris-Glycine acrylamide gels with 10 µg of the total *E. coli* protein per lane. After SDS-PAGE, polypeptides were either visualized by amido black staining or transferred onto a PVDF membrane (Bio-Rad Laboratories, USA) for Western blotting. Western blots were incubated in PBS containing 0.05% Tween-20 (PBS-T) and 10% nonfat dry milk to block nonspecific protein-binding sites. In all subsequent incubation steps, the blots were washed with PBS-T alone or incubated in PBS-T containing antibodies. Rabbit anti-EQPT antibody was used to detect SPANX-N and affinity-purified F(ab′)_2_ fragments of goat anti-rabbit IgG conjugated to horseradish peroxidase (HRP; Jackson ImmunoResearch, West Grove, PA) used as the secondary antibody. The HRP conjugates were visualized using TMB reagent according to the manufacturer's protocol (Kirkegaard & Perry Laboratories, Gaithersburg, MD). Because the peptide representing EQPT antibodies is conservative for SPANX-N proteins, it recognized all five SPANX-N proteins expressed in *E. coli* cells (Figures S1a and S1b). No cross-reactivity between SPANX-B, SPANX-C, and SPANX-N proteins was observed with the ANEA, SNEV, or EQPT antibodies by Western blot (Figures S2a and S2b). Pre-absorption of an antibody with the corresponding peptide used for immunization abolished the signal (data not shown).

### Western blot analysis in human tissues and cell lines

To analyze expression of the SPANX-N proteins in normal tissues and cancer cell lines, the cells were mixed with SDS sample buffer containing a protease inhibitor cocktail (Sigma-Aldrich Corp., St. Louis, MO, USA), homogenized and resolved in 4–20% Tris-Glycine acrylamide gel. Following electrophoresis, the proteins were transferred to PVDF membranes (Millipore, Billerica, MA, USA) for 40 min at 15 V in transfer buffer (50 mM Tris, 380 mM glycine, 0.1% SDS and 20% methanol) by the semi-dry method. All subsequent steps were carried out in PBS containing 0.05% Tween-20 (TPBS). After blocking for 30 min with 10% non-fat milk-TPBS, the membranes were exposed to 1/2500 diluted anti-EQPT and anti-alpha-tubulin antibodies (Sigma, St. Louis, Missouri, USA) for 1 h. Human anti-alpha-tubulin Abs were used as a positive, internal control. The PVDF membranes were washed three times with TPBS, incubated for 30 min with 1/2500 diluted HRP conjugated anti-rabbit IgG and anti-mouse IgG then washed as in the previous step. The membranes were incubated for 1 min with ECL plus reagents (GE Healthcare, Piscataway, NJ, USA). No bands were detected with the pre-immune serum. Pre-absorption of the EQPT antibodies with excess of the antigenic peptide (100 µM) abolished the signal (data not shown).

### Sperm preparation

Fresh semen samples were obtained from healthy men after informed consent using forms approved by the University of Virginia Human Investigation Committee. Ejaculates were allowed to liquefy at room temperature then counted using the computer-assisted sperm analysis system (Hamilton Thorne Research); only those that contained normal semen parameters were pooled for use in this study. The semen pool was recounted and an aliquot was diluted to 2×10^6^/ml in wash media (Nutrient Mixture F-10 HAM; Sigma) then fixed by adding a 16% solution of paraformaldehyde (Electron Microscopy Sciences, Ft. Washington, PA USA) to a final concentration of 3.2%. After a 10 min fixation at 4°C, the sperm were washed by centrifugation thrice with PBS then spotted onto slides and allowed to air-dry. For some experiments, motile spermatozoa were separated from seminal plasma, immature germ cells, and somatic cells (mainly white blood cells and epithelial cells) by the swim-up technique prior to fixation. The remaining semen pool was diluted 1∶5 in wash media; the spermatozoa were pelleted by centrifugation (500*g*), washed once in PBS and then frozen at −80°C until protein extraction was performed.

### Indirect immunofluorescent analysis of the sperm with anti-SPANX antibodies

Slides containing air-dried human spermatozoa were washed (3X) in PBS to rehydrate the cells, incubated in 100% methanol for 10 min to permeabilize the cells then washed again in PBS (3X) before blocking was performed. Non-specific binding was blocked by incubating the slides in PBS containing 10% normal goat serum (heat-inactivated at 55°C for 30 min; GibcoBRL, Invitrogen Corp, Grand Island, NY USA) for 30–60 min prior to incubation with the primary antibodies. Rabbit polyclonal anti-EQPT, anti-ANEA or anti-SNEV antibodies were used to detect specific SPANX-N, SPANX-B or SPANX-C staining, respectively. The slides were incubated with the primary antibodies for 60 min, washed thrice in primary antibody buffer (PBS containing 0.025% Tween-20 and 1.5% normal goat serum; PBST-NGS), and then incubated for 30 min with Fluorescein-labeled, AffiniPure F(ab′)_2_ fragment, goat anti-rabbit IgG (Jackson ImmunoResearch Laboratories, West Grove, PA USA) diluted 1∶500 in PBST-3% NGS. Thereafter, the slides were washed with PBS (4X), incubated with PBS containing 2% paraformaldehyde for 10 min, and washed again in PBS (2X) before being mounted with SlowFade Gold antifade reagent containing DAPI (Molecular Probes Invitrogen, Eugene, OR USA). Dual-fluorescent labeling of the human spermatozoa for both SPANX-N and SPANX-A/D proteins was performed by adding a mouse polyclonal ascites fluid raised against recombinant SPANX-A to the primary antibody reaction and CY3-labeled AffiniPure F(ab′)_2_ fragment, goat anti-mouse IgG/IgM (Jackson ImmunoResearch Laboratories, West Grove, PA USA) diluted 1∶1300 to the secondary antibody reaction. All incubations were performed at room temperature. Labeled cells were visuaslized with a Zeiss Axioplan 2 microscope equipped with a Hamamatsu digital camera.

### Immunostaining of human testis sections

Normal human testis sections were collected from formalin-fixed, paraffin-embedded samples (Cybrdi, Frederick, MD, www.cybrdi.com). Immunostaining for SPANX-N was obtained after heat unmasking (5 min at 90°C in standard citrate buffer using a temperature-controlled microwave) with overnight incubation of the primary antibody at 4°C. Secondary antibody was purchased from Invitrogen (Alexa Fluor series). The primary antibody was omitted in the incubation step for negative controls. Photographs were taken with a Deltavision system.

### Immunocytochemistry of tumor cells

Immunocytochemistry was performed on 938MEL cells fixed for 1 hour in Histochoice (Amresco Inc., Solon, Ohio, USA) and briefly heat-unmasked in standard citrate buffer. Incubation with the primary antibodies (EQPT antibody against SPANX-N and mouse anti-human Ki67 (BD-550609, Pharmingen, San Jose, CA USA), both at 1∶100 dilution was performed overnight at 4°C. EQPT antibody was omitted in the negative control. Secondary fluorochrome-conjugated antibodies were from the Alexa series (Invitrogen, Carlsbad, CA, USA).

### Amplification and sequencing of the *SPANX-N* genes

A total of 93 human individual genomic DNA samples were obtained from the Coriell Institute for Medical Research. A multibreed plate of 33 canine DNA samples was kindly provided by Dr. Mark Neff (University of California, Berkeley). The fragments containing *SPANX-N* sequences were PCR amplified from human and canine genomic DNA samples using a set of specific primers (Table S1). Sequence forward and reverse reactions were run on a 3100 automated Capillary DNA Sequencer (PE Applied Biosystems). DNA sequences were compared using the GCG DNA ANALYSIS Wisconsin Package (www.accelrys.com/support/bio/faqs_wis_pkg.htlm) and National Center for Biotechnology Information BLAST. Accession numbers of sequences are presented in Table S2.

### Electrophoretic mobility shift (EMSA) analysis

Overlapping fragments of *SPANX-N* and *SPANX-A/D* promoter regions were synthesized by PCR with the specific primers (Table S3). EMSA was performed as described earlier [16]. The luciferase control as well as 11 ZF DNA binding domain of CTCF protein were synthesized from the Luciferase T7 control DNA and pET16b-11ZF construct, respectively [17], [18], with the TnT reticulocyte lysate coupled *in vitro* transcription-translation system (Promega, Madison, WI, USA). Promoter-containing DNA fragments were ^32^P-labeled, gel purified, and used as DNA probes for gel mobility shift assays with equal amounts of *in vitro* translated luciferase and CTCF proteins as described [17], [18]. All the fragments were cloned into TA vector and sequenced before their analysis by EMSA. Binding reactions were carried out in buffer containing standard PBS with 5 mM MgCl2, 0.1 mM ZnSO4, 1 mM DTT, 0.1% NP40, and 10% glycerol in the presence of polydIdC and salmon sperm DNA. Reaction mixtures of 20 µl final volume were incubated for 30 min at room temperature and then analyzed on 5% nondenaturing PAGE run in 0.5× TBE buffer. For electrophoretic mobility gel-shift assay (EMSA) with *in vitro* methylated DNA probes, treatment with the *Sss*I-methylase was performed as previously described [16]. The extent of methylation was verified by digestion overnight with *Sau*96I restriction endonuclease.

### Sequence analysis

The *SPANX-N* homologue in the dog genome was detected by WISE2 searches (http://www.sanger.ac.uk/Software/Wise2/) using alignments of human and rodent *SPANX-N* copies as the profile. Sequences were aligned with Dialign2 [19; http://bibiserv.techfak.uni-bielefeld.de/dialign/ and MAVID [20; http://baboon.math.berkeley.edu/mavid/]. Conservation profiles were obtained using GeneDoc [21]. Phylogenetic trees were obtained by PAML v. 3.13 [22; http://abacus.gene.ucl.ac.uk/software/paml.html] after excluding all positions with gaps; the bootstrap values were calculated using PHYML v2.4.4 [23; http://atgc.lirmm.fr/phyml/] with default parameters.

### Isolation of *SPANX-A1* and *SPANX-A2* containing loci by TAR cloning in yeast

The TAR (transformation-associated recombination) cloning method is described in [24], [25]. Briefly, to isolate *SPANX-A1* and *SPANX-A2* containing loci, the vector TAR-A was constructed from basic vector pVC604. TAR-A contains 5′ *Sac*II/*Spe*I 156 bp and 3′ *Cla*I/*Spe*I 122 bp targeting sequences that were chosen and amplified from the available human genome sequence and flank the inverted copies of *SPANX-A1* and *SPANX-A2* in the human genome. The 5′ and 3′ targeting sequences correspond to positions 127,556–127,711 and 168,884–169,004 in BAC AL121881. The TAR vector was linearized with *Spe*I before TAR cloning experiments. Genomic DNAs from chimpanzee (*Pan troglodytes*), gorilla (*Gorilla gorilla*) and bonobo (*Pan paniscus*) (Coriell Institute for Medical Research, Camden, NJ) were used for TAR cloning experiments.

## Results

### *SPANX-N* homologs and their variation during early and more recent evolution of primates

**Figure 1:**
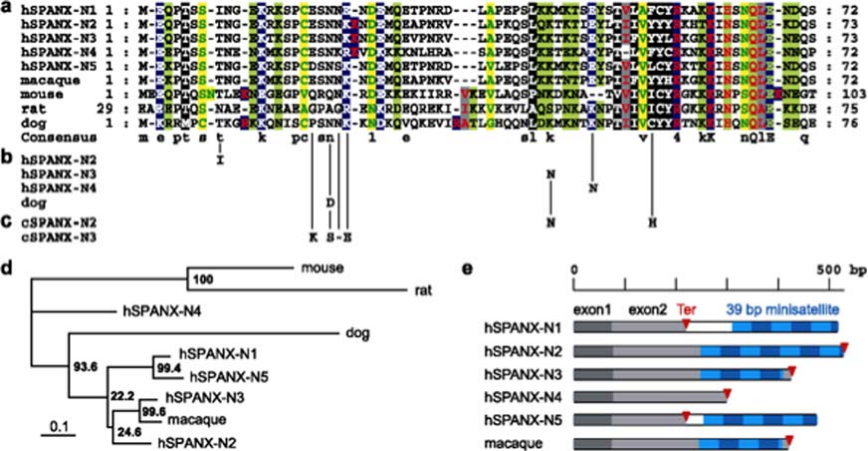
Comparison of mammalian SPANX-N proteins. (a) Alignment of the conserved core between human SPANX-N proteins and homologs in non-primate mammals. With the exception of rat SPANX-N, the core starts immediately at the N-terminus. The plot highlights physiochemical properties, and the sequence conservation is shown as a consensus at the bottom. (b) Polymorphic positions in the human and dog coding sequences (only core shown). Two nonsynonymous changes, N21D and S79N (not shown), were found in the four dog and two wolf alleles analyzed. The five compared SPANX-N2 alleles revealed a nonsynonymous change, T8I, and a synonymous polymorphism in codons 4, 80, and 151. One amino acid replacement, K43N, was found in the two compared SPANX-N3 alleles. The two analyzed SPANX-N4 alleles revealed only one nonsynonymous change, K48N. (c) Substitutions in chimpanzee SPANX-N2 and -N3 coding sequences compared to human homologs. Chimpanzee SPANX-N2 contains four nonsynomous substitutions, two of which are in the core (K43N and Y55H). In addition, there are three synonymous changes and a 65 aa long deletion caused by the deletion of five 39 bp minisatellite units. Chimpanzee SPANX-N3 contains 10 nonsynonymous changes compared to human SPANX-N3, with three in the conserved core (E18K, N21S, and K23E). There is also a single aa deletion, del22K, and two synonymous changes. (d) Phylogenetic relationship of SPANX-N proteins in mammals obtained using the maximum likelihood method. (e) Minisatellite variations in the C-terminal part of primate *SPANX-N* genes. With the exception of the human *SPANX-N4* locus, the C-terminal regions contain 39 bp minisatellite arrays (blue). In some cases, the translation termination codon (red) is located after the array, and the repeats encode the C-terminal portion of *SPANX-N* genes.

**Figure 2:**
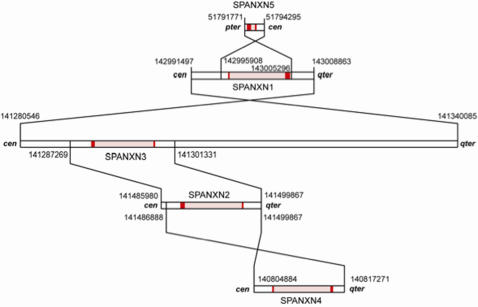
Evolution of *SPANX-N* genes. The probable scheme of evolution of the *SPANX-N* gene family is based on the pairwise alignments and breakpoint analysis. The exons are marked red in colour, intron regions are marked in pink. Positions relative to the centromere (cen) and telomere (qter) are shown. The numbers near duplication breakpoints indicate positions of the *SPANX-N* regions in the human genome (hg16; UCSC March 2006 genome version). The most likely scenario is that *SPANX-N3* was the original locus.

Previous studies suggest that the *SPANX* gene family is evolutionarily young and is in the process of expanding in hominoid species [13]. Here, bioinformatics and experimental approaches were used to identify more *SPANX-N* homologs in other species. A search for *SPANX-N* members in the draft chimpanzee genome (March, 2006) detected five contigs with incomplete sequences on the X chromosome. The complete coding regions of three of these chimpanzee homologs, *SPANX-N2*, *SPANX-N3*, and *SPANX-N5* were assembled by carrying out PCR against chimpanzee DNA using primers for human *SPANX-N* genes. The chimpanzee *SPANX-N* homologs encode proteins that share ∼95% identity with human SPANX-N proteins. A comparison of human and chimpanzee *SPANX-N2* coding sequences revealed four nonsynonymous substitutions, two of which are in the conserved core (K43N and Y55H) (Figure 1a and 1c). The chimpanzee *SPANX-N3* gene contains 10 nonsynonymous changes compared to human with three in the conserved core (E18K, N21S, and K23E). There is also a single deletion, del122K, and two synonymous changes. Similar to human, all chimpanzee *SPANX-N* genes, except *SPANX-N4*, contain 39 bp minisatellite repeats at their 3′ ends (Figure 1e). Because these repeats are also present in tamarin and rhesus *SPANX-N* genes (Figure S1), they likely arose during early primate evolution. *SPANX-N* genomic sequences were used to reconstruct the probable scheme of evolution of these genes in primates (Figure 2). Namely, that *SPANX-N3* was the original locus, and duplication of this chromosomal segment eventually produced gene clusters and gene subfamilies. Phylogenetic relationship of SPANX-N proteins in primates is shown in Figure S2.

Earlier a single *SPANX* copy was identified in mouse and rat genomes [13]. We screened the genome database in search of the canine *SPANX-N* locus. Two regions with significant similarity to human *SPANX-N* genes were identified in the canine genome; one of these regions is on the X chromosome and the other region is on chromosome 31. The X-linked canine *SPANX* gene is likely a pseudogene, because it has a stop codon in the middle of exon 2 and its expression is not detectable in testis by RT-PCR (data not shown). In contrast, the canine *SPANX-N* gene on chromosome 31 is expressed at a high level in testis (Figure S3); however, it encodes a protein that shares only 30% identity with human and mouse SPANX-N proteins. One *SPANX-N*-related gene and one pseudogene were also detected in the wolf genome. Sequence analysis of the *SPANX-N* gene in 33 canine breeds revealed four alleles that may be useful for pedigree analysis (Table S4A and Table S4B). Two nonsynonymous changes were found in four canine and two wolf alleles. Polymorphic positions in the human and canine coding sequences are shown in Figure 1b. Phylogenetic relationship of SPANX-N proteins in mammals is shown in Figure 1d.

**Figure 3:**
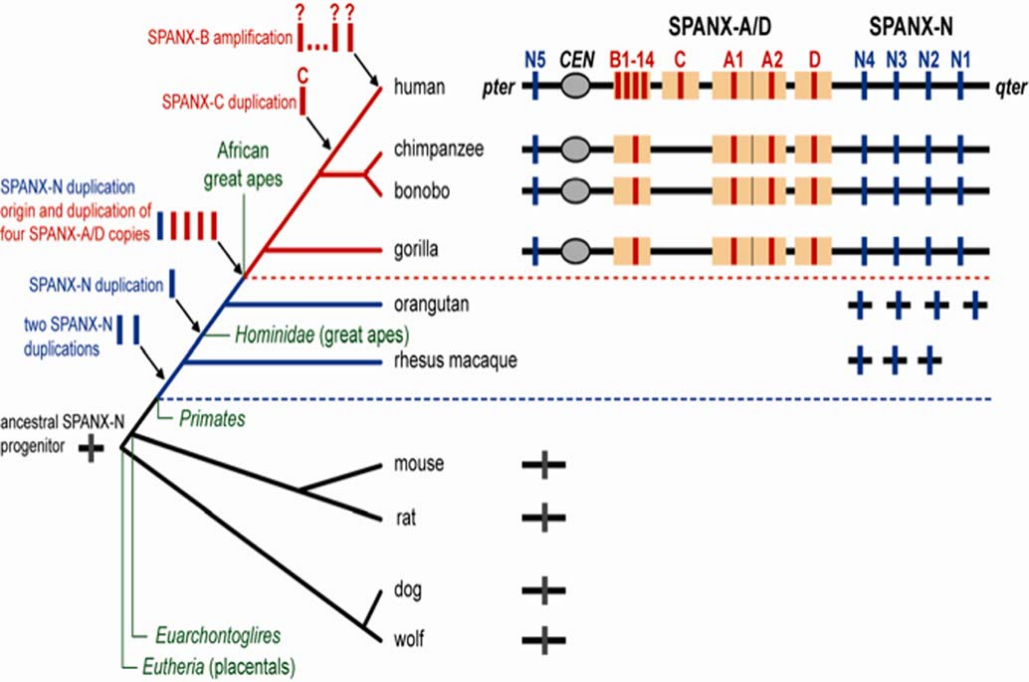
An evolutionary history of the *SPANX* gene family. The common ancestor of rodents, canine, and primates apparently had a single *SPANX-N* subfamily gene. An extensive search for potential divergent members of the *SPANX-N* family in the mouse, rat, dog and wolf genomes failed to detect any. A single *SPANX* gene is marked as a grey box. Orangutan and rhesus macaque have three and four copies of *SPANX-N* genes, correspondingly (boxes in blue). The emergence of the *SPANX-A*/*D* gene subfamily (boxes in red) is a more recent event, subsequent to the separation of the hominoid lineage from orangutan and rhesus macaque. Apparently, this subfamily evolved via duplication of one of the *SPANX-N* genes accompanied by deletion of the distal part of exon 2 (minisatellites) and rapid divergence. *SPANX-A/D* genes are impeded in segmental duplications (boxes in yellow). African Great Apes (bonobo, chimpanzee and gorilla) have four members of the *SPANX-A/D* subfamily. Notably, duplication of *SPANX-C* and amplification of *SPANX-B* genes from 1 to 14 copies appears to be human lineage specific.

A previous analysis of organization of the *SPANX-A/D* genes in African Great Apes has shown that two loci, *SPANX-B* and *SPANX-D,* are present in apes, but *SPANX-C* is human specific [5], [13]. A search for *SPANX-A* sequences in the chimpanzee genome draft (March, 2006) detected only one contig with a single *SPANX-A* sequence on the X chromosome, while in human there are two genes, *SPANX-A1* and *SPANX-A2*, organized as an inverted repeat. We isolated the *SPANX-A1/A2* synthenic regions from chimpanzee, gorilla and bonobo, as ∼50 kb DNA segments, using a TAR cloning technique (see Methods for details) and demonstrated that organization of this locus in African Great Apes is similar to human, i.e. the inverted repeats of the *SPANX-A1/SPANX-A2* genes embedded into segmental duplications. Collectively, the results presented here and published elsewhere [5], [13] allowed us to reconstruct the evolutionary history of the *SPANX* gene family in detail (Figure 3). The common ancestor of rodents, canine, and primates apparently had a single *SPANX-N* subfamily gene. Chimpanzee, orangutan and rhesus macaque have five, four and three copies of *SPANX-N* genes, correspondingly. The emergence of the *SPANX-A*/*D* gene subfamily is a more recent event, subsequent to the separation of the hominoid lineage from orangutan and rhesus macaque. Apparently, this subfamily evolved via duplication of one of the *SPANX-N* genes accompanied by deletion of the distal part of exon 2 (minisatellites) and rapid divergence. Notably, African Great Apes have four members of the *SPANX-A/D* subfamily, whereas duplication of *SPANX-C* and amplification of *SPANX-B* genes appears to be human lineage specific.

### Analysis of genetic variations in *SPANX-N* genes in human population

Previous studies revealed a high frequency of genetic variations in *SPANX-A/D* genes. Most of them resulted from gene conversion events between the genes [5]. In the present study we analyzed sequence variations in *SPANX*-*N1*, -*N2*, -*N3*, *-N4* and -*N5* genes from 93 normal human individuals. Exons 1 and 2 and flanking regions were PCR amplified using specific primers (Table S1), and the amplified DNA fragments were sequenced. The results are summarized in Table 1 and Table S2. Sequence analysis identified four alleles of *SPANX-N1*, five alleles of *SPANX-N2*, and two alleles each of *SPANX-N3*, *SPANX-N4* and *SPANX-N5*. None of the four *SPANX-N1* variants contained mutations resulting in amino acid substitutions. The five *SPANX-N2* alleles included one nonsynonymous change, T8I, in exon 1 and four synonymous substitutions in codons 4, 80, and 151. One amino acid replacement, K43N, in exon 2 was found in the two *SPANX-N3* alleles. The two *SPANX-N4* alleles revealed one nonsynonymous change, K48N in exon 2. All other variants had single synonymous missense mutations. SPANX-N5 variants had only synonymous mutations in exon 2. In addition, *SPANX-N1* and *SPANX-N5* genes keep a C to T mutation in exon 2 in all DNA samples analyzed, which causes generation of the premature stop codon. In contrast to the *SPANX-A/D* subfamily, none of the *SPANX-N* variants was due to gene conversion events. DNA sequence analysis revealed five, seven, four and five copies of the 39 bp minisatellite repeat in *SPANX-N1*, *SPANX-N2*, *SPANX-N3*, and *SPANX-N5*, respectively. In *SPANX-N1* and *SPANX-N5,* the minisatellite is located after the stop codon. In *SPANX-N2* and *SPANX-N3*, the minisatellite repeats are in frame with exon 2 and encode the C-terminus of the protein. DNA sequence analysis revealed no polymorphism in the *SPANX-N* minisatellites (data not shown).

**Table 1:** Nucleotide variations in the human *SPANX-N1*-, -*N2*, -*N3*, -*N4* and *-N5* genes

*SPANX-N1*	*SPANX-N2*	*SPANX-N3*	*SPANX-N4*	*SPANX-N5*
C/A (50%)*	G/C (2%)	**C/G** (18%)	**A/T** (2%)	G/C; T/C (59%)
552 nc	12 ex1	129 ex2	144 ex2	1016 ex2, 1091 nc
G/A (9.5%)	**C/T** (2%)			
292 nc	23 ex1			
CT/GT (2.5%)	C/A (16%)			
608, 609 nc	240 ex2			
	C/T (58%)			
	453 ex2			

* In parenthesis - the frequencies of sequence variants. Positions of nucleotide changes either in exon 1 or exon 2 (ex1 and ex2) are shown below the nucleotide changes.

nc – noncoding region.

In red – the nucleotide changes leading to amino acid substitutions.

Complete *SPANX-N* sequences are listed in Table S2.

To summarize, in contrast to the *SPANX-A/D* subfamily where frequent gene conversion events are a driving force of *SPANX-A/D* gene variantion in human populations [5], a normal polymorphism is characteristic for the evolutionary old *SPANX-N* subfamily. This difference may be explained by a higher level of divergence between *SPANX-N* genomic regions compared to the recently amplified *SPANX-A/D* loci.

### Development of SPANX-N specific antibodies

To determine the localization of SPANX-N proteins, we generated a polyclonal rabbit antibody EQPT against a chemically synthesized SPANX-N peptide (see Methods for details). Based on the peptide sequence chosen from the conservative *N*-terminus of SPANX-N3, the EQPT antibody should recognize at least three SPANX-N proteins (SPANX-N1, -N2, and -N3). To check a specificity of Abs, we expressed all five *SPANX-N* genes in *E. coli* cells. Western blot analysis with five recombinant SPANX-N proteins **s**howed that the affinity-purified anti-EQPT-antibody recognizes equally well all five individual SPANX-N proteins (Figures S4a and S4b). Apparent molecular weight of recombinant proteins expressed in *E. coli* cells is 13 kDa, 27 kDa, 23 kDa, 17 kDa and 13 kDa for SPANX-N1, -N2, -N3, -N4, and –N5, respectively (Figure S4c). Notably, mobility of SPANX-N proteins is approximately 6 kDa higher than that predicted from their coding regions (8 kDa, 20 kDa, 16 kDa, 11 kDa and 8 kDa for SPANX-N1, -N2, -N3, -N4 and –N5, correspondingly). A comparably slower mobility in acrylamide gels was previously described for SPANX-A/D proteins [3], [6] and is likely due to clustering of charged amino acid residues.

### SPANX-N proteins localize to the acrosome in spermatozoa

**Figure 4:**
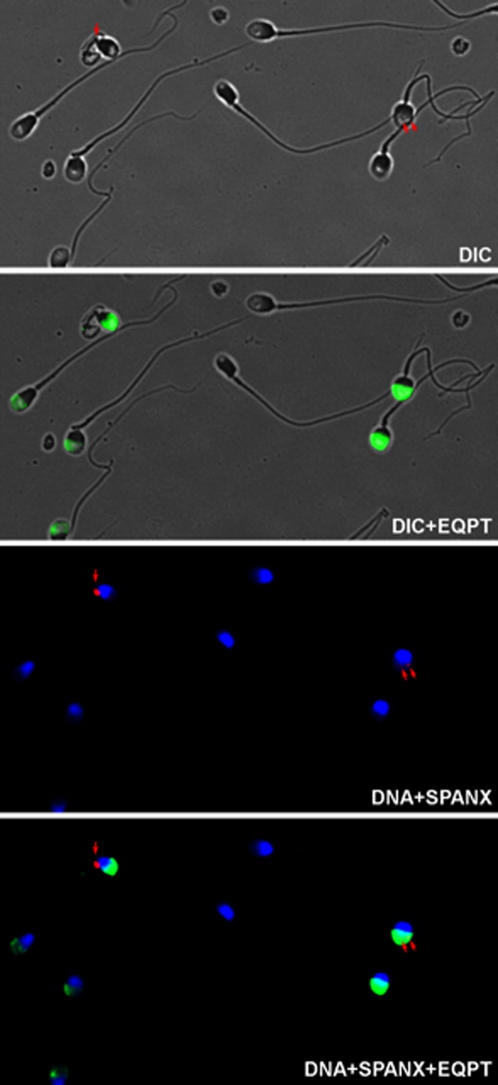
Indirect immunofluorescent staining of fixed, permeabilized swim-up spermatozoa with antibodies against SPANX-N proteins (in green). The SPANX-N immunofluorescence is observed in the acrosome of spermatozoa. The SPANX-A/D immunofluorescence is observed in association with nuclear craters in the cytoplasmic droplet at the posterior sperm head, or in both (red dots). DNA staining with DAPI is in blue.

Immunofluorescence localization revealed intense staining in the acrosome of formaldehyde-fixed, methanol-permeabilized, human spermatozoa with postimmune EQPT antisera and the affinity purified antibodies (Figure 4). Spermatozoa incubated with preimmune sera or with EQPT peptide exhibited no fluorescence. These data indicate that the *SPANX-N* genes encode acrosomal proteins. Their localization is different from that of SPANX-A/D proteins based on immunostaining with previously reported polyclonal antibodies [6]. Because specificity of the polyclonal antibodies to individual SPANX-A/D isoforms was not determined, we repeated the immunostaining of spermatozoa with newly developed polyclonal antibodies, SNEV and ANEA, specific to SPANX-C and SPANX-B proteins, respectively (see Methods for details). Our results confirmed localization of SPANX-B and SPANX-C proteins in the nuclear craters and cytoplasmic droplets (Figure S5c). It is worth noting that the EQPT antibody detects SPANX-N proteins in greater than 90% of human spermatozoa. In contrast, staining with SNEV and ANEA antibodies confirmed the previous observation that SPANX-A/D proteins are present in only half of ejaculated spermatozoa [4], [6]. Thus, rapid evolution and expansion of the *SPANX* gene family resulted in generation of two classes of proteins with presumably different functions in spermatogenesis.

### SPANX-N are expressed postmeiotically

**Figure 5:**
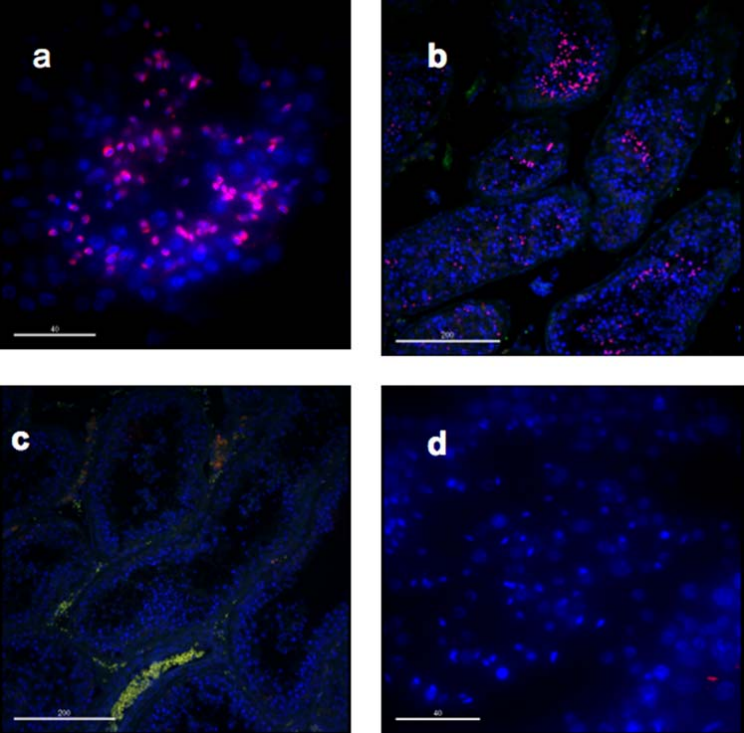
Immunostaining of SPANX-N (a, b) and the negative control (c, d) at low (a, c) and high magnification (b, d) on normal human testis sections (bars indicated). The staining is clearly specific for late spermatids and spermatozoa (in pink, a, b). Autofluorescence (i.e., non-specific signal, a, b) is also detectable in red blood cells and can be recognized by the overlapping signals in the green and red wavelengths. Nuclei are counterstained with DAPI (in blue).

To detect the stage specificity of *SPANX-N* gene expression, we performed localization of SPANX-N on normal human testis sections. The staining was positive only in late spermatids and spermatozoa within seminiferous tubules (Figures 5a and 5b) and is specific, as indicated by its absence in the negative controls (Figures 5c and 5d) both at low (Figures 5b and 5c; bar: 200 um) and high magnification (Figures 5a and 5d; bar: 40 um). These data suggest that SPANX-N protein translation occurs postmeiotically, as observed for SPANX-A/D [6], [26].

**Figure 6:**
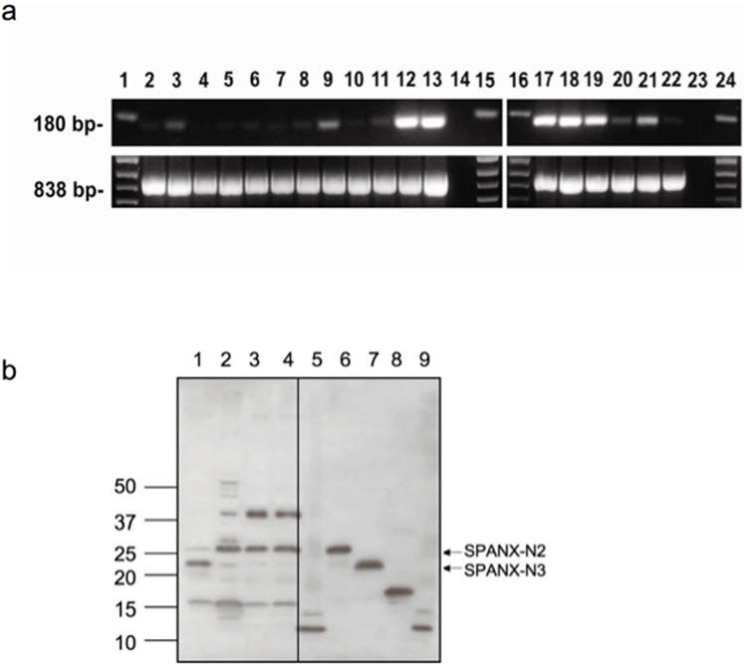
(a) RT-PCR analysis of the *SPANX-N* gene subfamily in normal adults tissues and cancer cell lines. cDNA was prepared from a panel of human tissue mRNAs and cell lines. Oligonucleotides were designed within exons 1 and 2 to amplify putative transcripts. The observed bands of the expected size 180 bp were sequenced and confirmed to correspond to *SPANX-N* genes. The strongest expression of *SPANX-N* genes was observed in the normal testis (lanes 12 and 17) and in the LOXIMV1 melanoma cell line (lanes 13 and 18). Lanes 2–11 correspond to normal and tumor pairs of breast, cervix, prostate, lung and ovary; lanes 19–21 correspond to 938MEL, 888MEL, SKMEL28 melanoma cell lines; lane 22 corresponds to the SKOV3 ovarian cell line; lanes 1, 15, 16, 24 – ladder; lanes 14 and 23 - water. The cDNA templates used were normalized using actin, as shown at the bottom of the panel. (b) Western blot analysis of lysates from normal tissues using an anti-EQPT antibody. Lane 1 - lung, lane 2 - testis, lane 3 - placenta and lane 4 – prostate. Lanes 5–9: full-size SPANX-N proteins expressed in the pET-11d in Bl21 cells. The mobility of SPANX-N proteins produced in *E. coli* cells is 13 kDa for SPANX-N1, 27 kDa for SPANX-N2, 23 kDa for SPANX-N3, 17 kDa for SPANX-N4 and 13 kDa for SPANX-N5.

### *SPANX-N* genes are transcribed in normal nongametogenic tissues

Previously it was shown that expression of *SPANX-A/D* genes is restricted to the normal testis and certain tumors [2]–[4]. In the present study, expression of *SPANX-N* genes was examined in normal adult tissues as well as a variety of tumor specimens and tumor lines.

Expression of *SPANX-N* genes was examined in eighteen normal tissues by RT-PCR with a pair of primers that recognize all five *SPANX-N* genes (Table S1). The predicted size of spliced *SPANX-N* transcripts was not detected in brain, liver, skeletal muscle, spleen, heart, lymphoma, lymph node, and kidney. Unexpectedly, in addition to expression in the testicular tissue, qualitative RT-PCR showed a weak expression of *SPANX-N* in breast, cervix, prostate, lung, ovary, placenta, proximal and distal colon, stomach, and uterus. Quantification of gene expression revealed that the expression level of *SPANX-N* mRNA in these tissues was 50–100 times lower than that in testis. *SPANX-N* expression in several nongametogenic tissues (breast, cervix, prostate, lung and ovary) is shown in Figure 6a (lanes, 2, 4, 6, 8 and 10). The coding regions have a 180 bp nucleotide sequence specific for each *SPANX-N* gene. Cloning of RT-PCR products into a TA vector and sequencing of the inserts from individual colonies allowed us to verify the specificity of a PCR reaction and to clarify if all five *SPANX-N* gene family members or only some are expressed in a certain tissue. The pattern of expression of *SPANX-N* genes seems to be different in different tissues. All five transcripts were detected only in testis. In most *SPANX-N* positive tissues, only one or two *SPANX-N* members are predominantly expressed (Table 2). To conclude, in contrast to *SPANX-A/D* that exhibits testis-specific expression, *SPANX-N* genes are expressed at a low level in a variety of normal adult tissues.

**Table 2:** RT-PCR analysis of *SPANX-N* expression in normal and tumor tissues and cell lines

Tissues/Cell lines	*SPANX*				
	*N1*	*N2*	*N3*	*N4*	*N5*
*Normal tissues*					
Testis	+	+	+	+	+
Prostate	−	+	+	+	+
Cervix	+	+	−	−	−
Placenta	−	+	−	−	+
Lung	−	−	+	−	−
Distal colon	−	−	+	−	+
Proximal colon	−	−	−	−	+
Brain	−	−	−	−	−
Liver	−	−	−	−	−
Skeletal muscle	−	−	−	−	−
Spleen	−	−	−	−	−
Heart	−	−	−	−	−
Kidney	−	−	−	−	−
Lymphoma	−	−	−	−	−
Lymph node	−	−	−	−	−
*Primary uterine tumors*					
Tumor 2952	+	−	−	−	−
Tumor 3017	+	−	−	−	+
Tumor 3047	+	−	+	−	−
*Tumor cancer cell lines* *					
SKOV3	+	−	+	−	+
HeLa	+	−	+	+	−
*Melanoma cell lines*					
LoxIMV1	+	−	−	−	−
537 MEL	+	−	−	−	−
938 MEL	+	−	+	−	−
888 MEL	+	−	+	−	−

* SKOV3- ovarian; HeLa-cervix. Each RT-PCR product was cloned in TA vector. From 5 to 40 individual *E. coli* clones obtained for each sample were analyzed by sequencing. Cloning and sequencing of RT-PCR products determined the gene-specific transcripts in each tissue or cell line. 95 of 103 sequenced clones from different tumor tissues and cancer cell lines corresponded to *SPANX-N1*. Four of 120 sequenced clones from normal tissues corresponded to *SPANX-N1*. Expression of SPANX-N2 and SPANX-N3 proteins in lung, testis, placenta and prostate was confirmed by Western blot (Figure 6b).

### Expression of SPANX-N proteins in normal nongametogenic tissues

Using a polyclonal rabbit antibody against the synthetic peptide EQPT, expression of SPANX-N proteins was examined in nongametogenic tissues. Figure 6b shows Western blot analysis of protein extracts from several human normal tissues that were identified as positive for *SPANX-N* transcripts by RT-PCR (lung, testis, prostate, and placenta). In each tissue, the affinity-purified EQPT antibody detects at least one band, the mobility of which corresponds to that of the SPANX-N protein expressed in *E. coli* cells. Two bands on Western blots (27 kDa and 23 kDa) co-migrate with the SPANX-N2 and SPANX-N3 recombinant proteins. Additional bright bands on the Western blot (16 kDa and 40 kDa) are probably due to post-translational modification of the SPANX products or to protein complexes. Notably, similar extra-bands were previously detected by Western blot analysis of SPANX-A/D proteins in cancer cells [3], [6], [26]. Therefore, it is possible that there are common steps in post-translational modification of SPANX proteins. Bands with the mobility of 13 kDa and 17 kDa (corresponding to SPANX-N1, SPANX-N5 and SPANX-N4 proteins) were not observed. A failure to detect an immunoreactive band of 13 kDa is likely to indicate that SPANX-N1 and SPANX-N5 proteins are not translated at all. This is supported by the presence of a frameshift mutation in the exon 2 sequence of these genes that generates a premature STOP codon that may destabilize mRNA by nonsense-mediated mRNA decay (NMD), as has been shown for some other mRNA [27]. Therefore, these two genes are likely pseudogenes. A lack of the band of 17 kDa corresponding to SPANX-N4 may be due to a low level of the protein or its instability in the analyzed tissues. Thus, at least two proteins, SPANX-N2 and SPANX-N3, are expressed in some nongametogenic tissues.

### Expression and localization of SPANX-N in cancer cells

**Figure 7:**
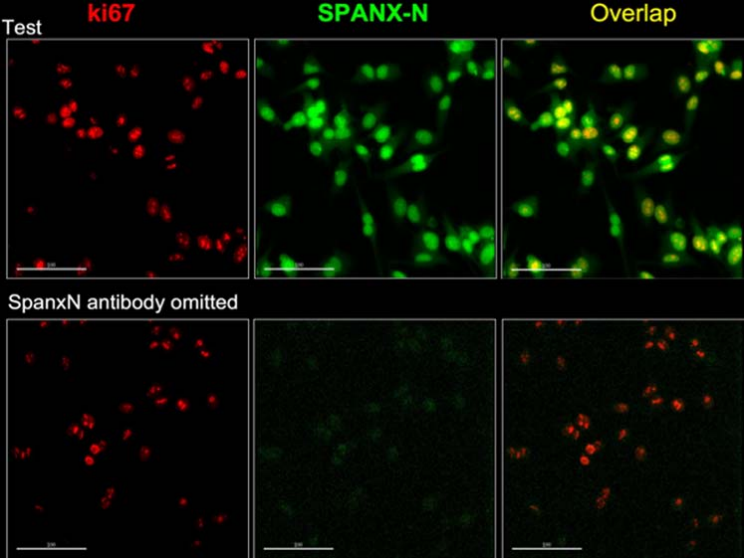
Immunocytochemical detection of SPANX-N in the melanoma cell line. 938MEL melanoma cell line (green; middle and right panels); co-immunostaining for the cell multiplication marker Ki67 (red; left and right panels; overlap with SPANX-N appears as yellow). Upper panels show the test samples, whereas bottom panels provide the negative control for SPANX-N (primary antibody omitted).

Expression of *SPANX-N* genes was also examined in cancer specimens and cancer cell lines. In total, 18% of primary ovarian and uterine cancers and 51% of the cancer cell lines analyzed (melanoma, ovarian, endometrial, colorectal, prostate, lung, cervix and breast) were positive for the presence of *SPANX-N* transcripts (data not shown). Intensity of PCR products was found to be heterogeneous, and some specimens yielded only faint amplicon bands. These were scored positive if the results could be reproduced by a repeated RNA extraction and specific PCR from the same tumor specimen resulting in clear bands. *SPANX-N* m RNA expression in breast, cervix, prostate, lung and ovary cancer tissues is shown in Figure 6a (lanes 3, 5, 7, 9 and 11). The highest level of *SPANX-N* expression comparable with that in testis was detected in some melanoma cell lines (for example, LoxIMV1 and 938MEL) (Figure 6a). Interestingly, 92% of the sequenced RT-PCR products from different tumors and cancer cell lines corresponded to *SPANX-N1* while only 3.3% of the clones from normal tissues corresponded to *SPANX-N1* (mostly in testis), suggesting preferable expression of this gene in malignant tissues (Table 2). The differential activation of *SPANX-N1* in cancer tissues suggests that it might be a new diagnostic marker for cancer. To examine the localization of SPANX-N proteins in tumor cells, immunocytochemical analysis was performed on the 938MEL melanoma cell line. Immunocytochemistry detected homogeneous SPANX-N expression (Figure 7, green) in the nucleus and cytoplasm of all cells independently of the cell cycle (as indicated by variable degree of co-immunostaining with Ki67) (Figure 7, red). Thus, the localization of SPANX-N in cancer cells is congruent with that of SPANX-A/D [3]. We also addressed a question of whether expression of *SPANX-N* genes in cancer cells correlates with activation of S*PANX-A/D* genes. Analysis of three melanoma cell lines (938MEL, 537MEL and LoxIMV1), *SPANX-N* positive, revealed the presence of *SPANX-A/D* transcripts also (data not shown).

### Analysis of promoter sequences of *SPANX-N* and *SPANX-A/D* genes

To shed light on the potential mechanism of differential expression of the *SPANX-A/D* and *SPANX-N* gene subfamilies in nongametogenic tissues, we carried out a detailed analysis of highly conserved 5′ UTR noncoding sequences.

**Figure 8:**
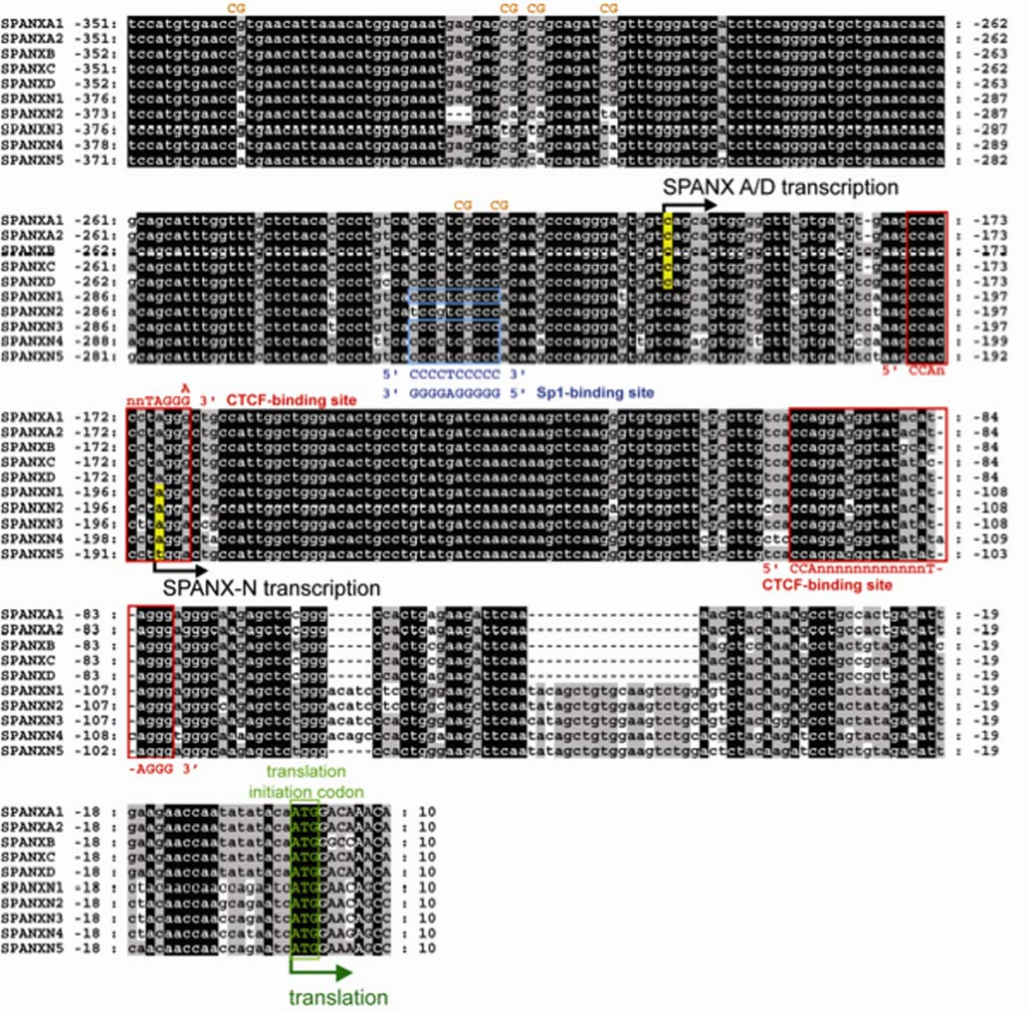
Comparison of human *SPANX-A/D* and *SPANX-N* promoters. Detected transcription starts are marked in yellow, the translation initiation codons ATG are marked in green. Noncoding sequences are in lowercase. *SPANX-N* copies differ from *SPANX-A/D* genes by the almost complete lack of all CpG dinucleotides in the promoter regions (in orange); however, these CpGs are perfectly preserved in all of the *SPANX-A/D* copies. CpG sites are marked only on one strand. Sp1 binding consensus in four *SPANX-N* copies is marked in blue. CTCF-binding sites are marked by red boxes.

Here, the transcription start points for the *SPANX-N* and *SPANX-A/D* genes were determined by RT-PCR using a set of nested primers (Table S1). For *SPANX-N* and *SPANX-A/D* genes, transcription starts at -193 and -204 nucleotides from the initiation codon, respectively (Figure 8). Our mapping results are in agreement with the recent work of Wang and co-authors [28] who identified the *SPANX-B* promoter region using a functional test. Further sequence analysis indicates that *SPANX-A/D* promoters include 16 CpG dinucleotides, 14 of which are mutated within *SPANX-N* promoters (Figure 8). Given the well-known link between CpG island methylation and gene expression [29]–[31], it is possible that the presence or absence of CpG dinucleotides may influence patterns of *SPANX-A/D* and *SPANX-N* genes in nongametogenic tissues. We suggest that methylation-mediated inactivation of *SPANX-A/D* genes is more efficient compared to *SPANX-N* genes. The Sp1-binding site found within the promoter sequence of four *SPANX-N* genes (Figure 8) may result in an even greater difference in promoter methylation because binding of the Sp1 transcription factor may prevent DNA methylation [32], [33]. Collectively, these observations suggest that expression of the *SPANX* gene family is generally regulated through promoter demethylation. The evolutionary old group of genes, *SPANX-N*, may partially escape this regulation possibly due to a lower density of CpG dinucleotides that leads to transcription of these genes in some nongametogenic tissues.

### CTCF binds to promoter regions of *SPANX-N* and *SPANX-A/D* genes

**Figure 9:**
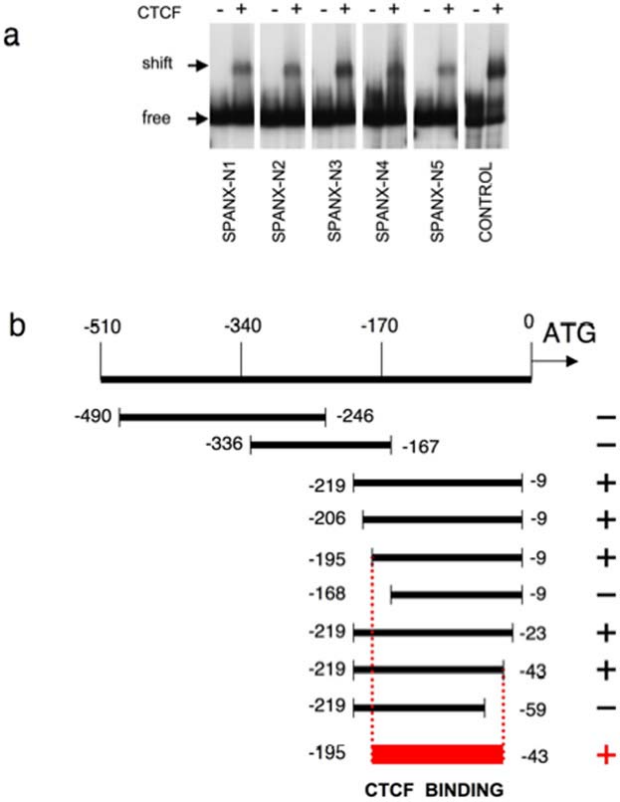
*In vitro* interaction of CTCF with the promoter sequences of *SPANX* genes. (a) EMSA was carried out with either control lysate (−) or lysate containing the *in vitro* translated 11 ZF DNA binding domain of CTCF protein (+). The positions of the bound CTCF-DNA complexes, containing the 11ZF domain, are indicated on the left by arrow (shift). Free DNA probe is also indicated (free). Control: a positive control (c-myc promoter) of the EMSA reaction. (b) A schematic representation of the overlapping fragments of the *SPANX-N* promoter DNA sequences used in EMSA. The −195 to −43 bp is the smallest fragment showing retarded migration (red box). The ATG is considered as +1. On the right is CTCF binding of the fragments (+ positive; − negative).

To elucidate a molecular mechanism of activation of *SPANX-A/D* and *SPANX-N* genes in testis and a complete or partial block of transcription in nongametogenic tissues another experiment was carried out. Recent publications indicate that reciprocal binding of the transcriptional factors, CTCF or BORIS, to a promoter sequence may be a general mechanism of regulation of the cancer-testis (CT) specific genes, expression of which is restricted to male germ cells [34]–[36]. CTCF and BORIS genes encode for 11 zinc-finger DNA binding proteins that recognize the same target sequence but exhibit different expression profiles [37]–[39]. CTCF protein is expressed in nongametogenic tissues and its binding to a promoter of CT genes induces a transcription silencing. BORIS is expressed exclusively during spermatogenesis and functions as a transcriptional activator of cancer-testis (CT) genes. We explored the possibility that the promoter regions of *SPANX* genes contain CTCF/BORIS-binding sites. *In vitro* binding of the promoter fragments of *SPANX-N* and *SPANX-A/D* genes to the full-length CTCF protein was tested by EMSA. The promoter fragment corresponding to −195 to −43 bp common to both subfamilies was positive for CTCF-binding (Figures 9a and 9b). Mobility shifts were not observed with other promoter regions tested (for example, with the fragments corresponding to positions −490 to −246 bp or −336 to −167 bp from the ATG codon in the promoter sequences) (Figure 9b). These results demonstrated that CTCF binds *in vitro* to promoter regions of *SPANX* genes upstream of a translation start site. EMSA experiments also demonstrated that binding of CTCF to *SPANX* promoter sequences *in vitro* is not inhibited by methylation (data not shown) as it was shown for other genes with exclusive expression in testis [35], [36], [40], [41]. Detection of CTCF binding sites in the promoter region of *SPANX* genes suggests that their regulation is similar to that for other genes with a preferential expression in male germ cells [34]–[36]. Taken together, CTCF/BORIS binding and a lower density of CpG dinucleotides in the promoter region of *SPANX-N* genes provide a reasonable explanation of activation of these gene subfamilies in testis and their differential expression in nongametogenic tissues.

## Discussion

Genes involved in reproduction have long been noted to evolve relatively rapidly (for example in [13], [42]–[45]), often through the agency of duplication and divergence [46]. Our analysis of *SPANX-N* gene evolution, variation, and expression provide a prime example. For example, SPANX-N proteins are targeted to the acrosomal region, suggesting that they may play a particular role in fertilization [47]. By contrast, the *SPANX-A/D* genes derived from them evolutionarily encode nuclear envelope proteins in spermatozoa, with a presumably altered function.

In addition to SPANX-N, several other acrosomal antigens have been identified [48]. They include SPAG9 [49], SP-10 [50], SAMP32 and SAMP 14 [51]–[53], ESP [53] and SPAM1 [54]. Only SPAM1 has a suggested role, in secondary zona binding. For SPANX-N, the existence of several expressed genes complicates functional studies. If there is a major conserved function, it could likely be identified in rodents, which have only a single *SPANX* gene, using knockout and co-immunoprecipitation technologies; but there would be no available models to look for the effects of selective ablation of the *SPANX-A/D* and additional *SPANX-N* genes in primates.

This study also shows that in contrast to *SPANX-A/D*, *SPANX-N* genes are expressed, though at low levels, in several nongametogenic tissues, including placenta, prostate, colon, cervix, stomach, uterus and lung. Sequencing of cDNA clones and Western blot analysis revealed that at least two gene family members, *SPANX-N2* and *SPANX-N3*, are expressed in proteins in these tissues as well as testis. It remains possible that SPANX-N proteins have some functions there, but the low expression levels may instead only represent leakage of transcription expression.

The expression pattern of *SPANX* genes may be related to the action of CTCF at its binding site in the promoter. CTCF binding in promoter regions is usually associated with CpG methylation and gene silencing [37], [38], as in the *MAGE-A1* and *NY-ESO-1* genes that also exhibit testis-specific expression [35], [36]. The somewhat higher expression of *SPANX-N* genes compared to *SPANX-A/D* genes in nongametogenic tissues could correlate with their lower level of CpG dinucleotides. In addition, *SPANX-N* promoters contain a potential recognition site for the transcription factor Sp1 that can protect CpG islands from *de novo* methylation [32], [33] leading to lesser gene silencing. As for the specific activation of *SPANX* genes in spermatogenesis, it is likely to be linked to demethylation of the entire promoter. The activation could be mediated by the programmed replacement of CTCF in the testis by its testis-specific paralogue BORIS [35], [36].

In contrast to restricted expression in other tissues, *SPANX-N* transcripts were found in a wide range of tumors. Dysregulation of *SPANX-N* in malignant tissues is intriguing because the *SPANX* gene cluster is co-localized with two cancer susceptibility loci: *TGCT,* encoding a testicular germ line cell tumor susceptibility gene [55], and *HPCX,* encoding a susceptibility gene for familial prostate cancer [56]. Regardless of a possible link between *SPANX-N* and carcinogenic process, their expression pattern infers that *SPANX-N* are CT (cancer testis) antigen genes. There is no explanation at present of the enrichment of CT genes on the X chromosome, where 22 of the 44 distinct reported families map [57]. It remains to be seen if its relatively specific association with tumors makes *SPANX-N1* a useful diagnostic marker to distinguish between normal and neoplastic tissues, or if it has any mechanistic connection to carcinogenesis that would make it a conceivable target for immunotherapy.

## Supporting Information

Figure S1 Alignment of primate SPANX-N proteins. The chimpanzee SPANX-N3 contains 4 minisatellite repeats in frame. The chimpanzee SPANX-N2 contains 2 minisatellite units while human SPANX-N2 contains seven 39 bp units. The chimpanzee SPANX-N5 has 5
(6.19 MB TIF)

Figure S2 Phylogenetic relationship of SPANX-N proteins in primates. The tree topology was obtained using PHYML v2.4.4 using default parameters and 100 replicates.
(0.32 MB TIF)

Figure S3 RT-PCR analysis of the canine SPANX-N expression. cDNA was prepared from the testis tissue using oligonucleotides designed within exons 1 and 2 to amplify a putative transcript. A 271 bp band of the expected size was observed. DNA sequenc
(0.37 MB TIF)

Figure S4 EQPT antibodies recognize all five SPANX-N isoforms.
(0.56 MB TIF)

Figure S5 Immunostaining of human spermatozoa with affinity-purified anti-EQPT, ANEA and SNEV antibodies.
(1.09 MB TIF)

Table S1 Primers used for amplification of the SPANX genes and their expression analysis
(0.07 MB DOC)

Table S2 Accession numbers of SPANX-N sequences
(0.05 MB DOC)

Table S3 Primers used for EMSA analysis
(0.03 MB DOC)

Table S4 Nucleotide and amino acid variants in the canine SPANX-N coding region
(0.07 MB DOC)
