# Supplementary figures and images for: Evolutionary Diversification of SPANX-N Sperm Protein Gene Structure and Expression
Source: PLoS One. 2007 Apr 4;2(4):e359. doi: 10.1371/journal.pone.0000359 (PMC1831492; doi:10.1371/journal.pone.0000359)

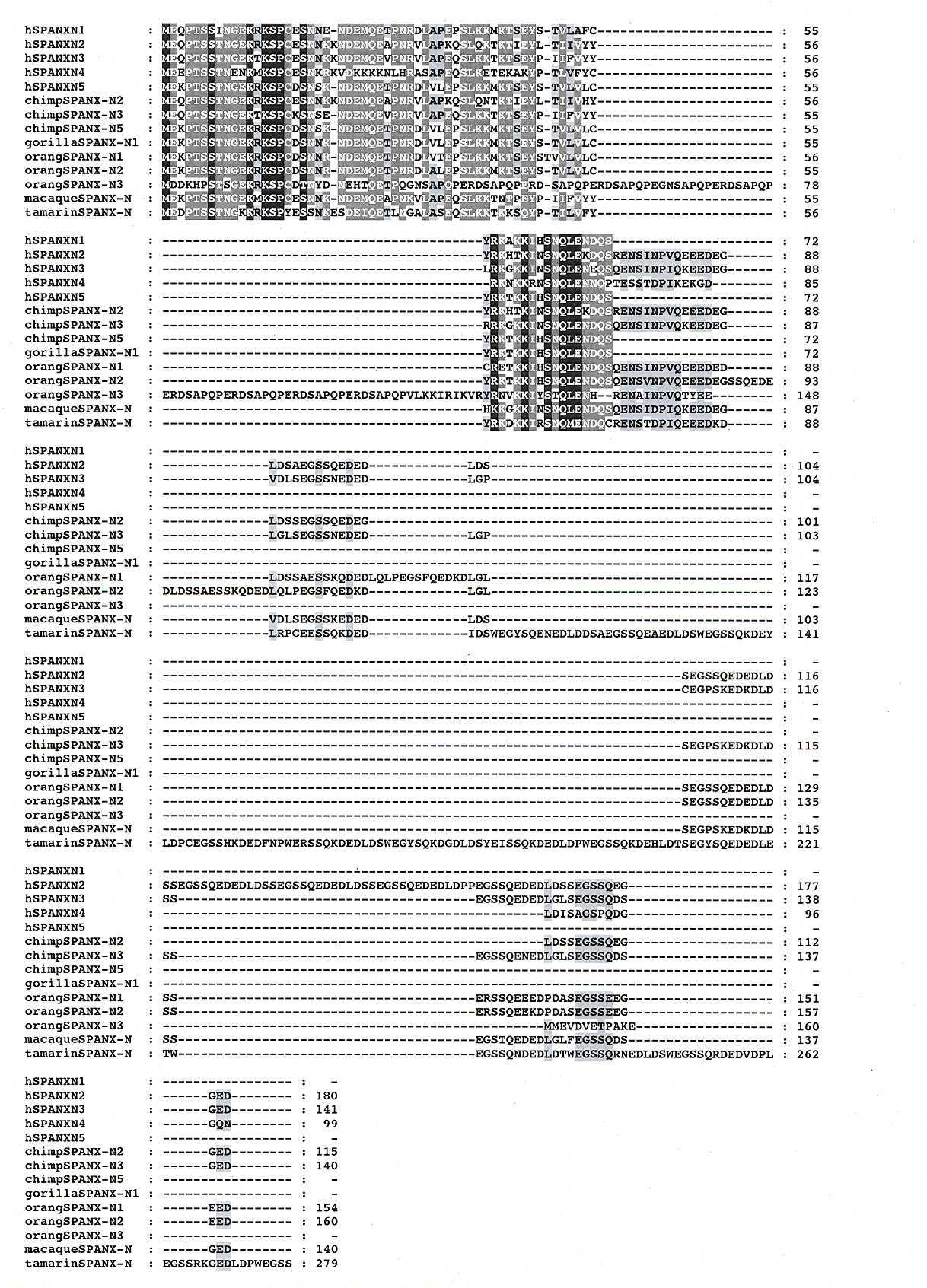

Supplement: Figure S1 — Alignment of primate SPANX-N proteins. The chimpanzee SPANX-N3 contains 4 minisatellite repeats in frame. The chimpanzee SPANX-N2 contains 2 minisatellite units while human SPANX-N2 contains seven 39 bp units. The chimpanzee SPANX-N5 has 5 (6.19 MB TIF) [file pone.0000359.s001.tif]

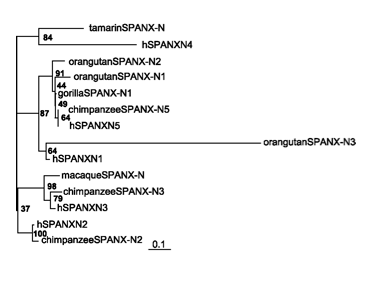

Supplement: Figure S2 — Phylogenetic relationship of SPANX-N proteins in primates. The tree topology was obtained using PHYML v2.4.4 using default parameters and 100 replicates. (0.32 MB TIF) [file pone.0000359.s002.tif]

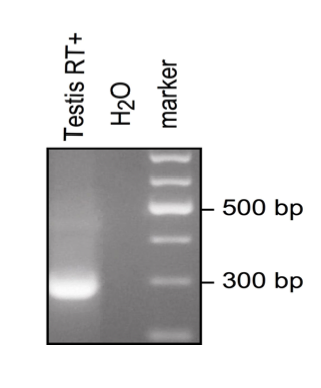

Supplement: Figure S3 — RT-PCR analysis of the canine SPANX-N expression. cDNA was prepared from the testis tissue using oligonucleotides designed within exons 1 and 2 to amplify a putative transcript. A 271 bp band of the expected size was observed. DNA sequenc (0.37 MB TIF) [file pone.0000359.s003.tif]

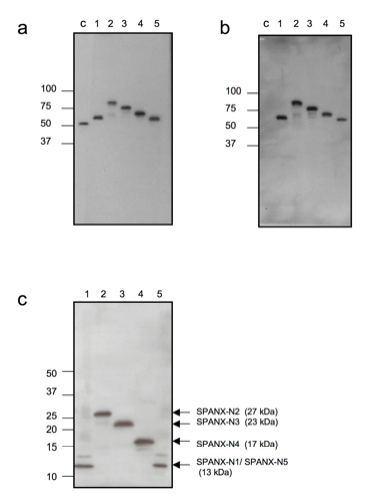

Supplement: Figure S4 — EQPT antibodies recognize all five SPANX-N isoforms. (0.56 MB TIF) [file pone.0000359.s004.tif]

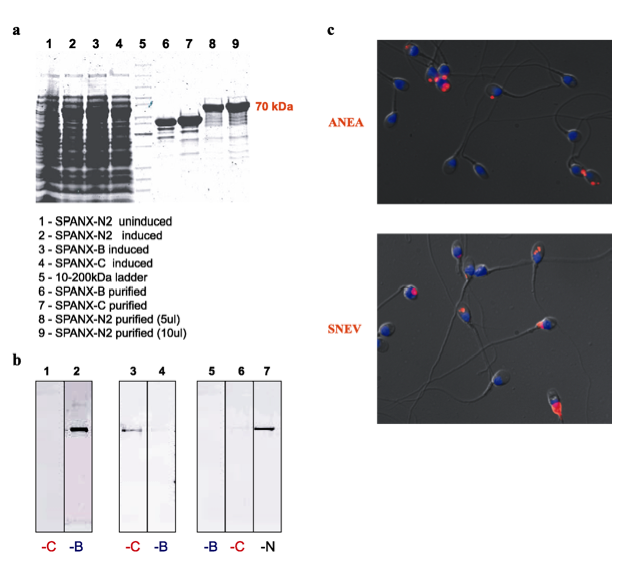

Supplement: Figure S5 — Immunostaining of human spermatozoa with affinity-purified anti-EQPT, ANEA and SNEV antibodies. (1.09 MB TIF) [file pone.0000359.s005.tif]
