# Supplementary material for: Evolutionary Diversification of SPANX-N Sperm Protein Gene Structure and Expression
Source: PLoS One. 2007 Apr 4;2(4):e359. doi: 10.1371/journal.pone.0000359 (PMC1831492; doi:10.1371/journal.pone.0000359)
Supplement: Table S1 — Primers used for amplification of the SPANX genes and their expression analysis (0.07 MB DOC) [file pone.0000359.s006.doc]

# Table S1. Primers used for amplification of the *SPANX* genes and their expression analysis

| **RT-PCR expression of *SPANX-C*, *SPANX-B* and *SPANX-N*** | |
| --- | --- |
| C-F/C-R (291 bp) | 5’-atggacaaacaatccagtgc-3'/5'-ctttgcaggtatttcaaccat-3' |
| B-F/B-R (309 bp) | 5'-atgggccaacaatccagtgt-3'/5'-ctttttaggtctttcagtcgt-3' |
| N1/N5-F/N1/N5-R (180 bp)  N1a-F/ N1a-R (216 bp)  N2a-F/ N2a-R (540 bp)  N3a-F/ N3a-R (423 bp)  N4a-F/ N4a-R (297 bp)  N5a-F/ N5a-R (216 bp) | 5’-aagaggaagagcccctgtga-3’/5’-ggtcattctccagttgatttga-3’  5’-taatgcccatggaacagcccacttcaagc-3’/5’-  taatgcctcgagtctcaggactggtcattctcc-3’  5’-taatgcccatggaacagccgacttcaagc-3’/5’- taatgcctcgagctagtcctccccaccctcctg-3’  5’-taatgcccatggaacagccaacttccagc-3’/5’- taatgcctcgagctaatcctccccactgtcctg-3’  5’-taatgcccatggaagagccaacttccagc-3’/5’- taatgcctcgagctaattctgcccaccatcctg-3’  5’-taatgcccatggaacagcccacttcaagc-3’/5’- taatgcctcgagtctcaggactggtcattctcc-3’ |

|  |  |
| --- | --- |
| **Site of initiation of transcription of *SPANX-N*** |  |
| 225-F (458 bp) | 5’- gcagtggtgctttgtgatgt-3’ |
| 209-F (442 bp) | 5’-atgtctaagccaccctagga-3’ |
| 206-F (439 bp): | 5’-tctaagccaccctaggactg-3’ |
| 198-F (431 bp): | 5’-accctaggactgccattg-3’ |
| 193-F (426 bp): | 5’-aggactgccattggctgg-3’ |
| 178-F (411 bp) | 5’- tgggacactgcctgtatgat-3’ |
| 130-F (363 bp) | 5’–cttgtcaccaggagggtata-3’ |
| 70-F (303 bp) | 5’-cttcaacatagctgtggaagt-3’ |
|  |  |
| N2-R | 5’-atggagttctcttgggactg-3’ |
|  |  |
| **Site of initiation of transcription of *SPANX-A/D*** |  |
| 291-F (468 bp) | 5'-atgcatcttcaggggatgct-3' |
| 251-F (418 bp) | 5'-tttgctctacacccctgtca-3' |
| 204-F (371 bp) | 5'-agcagtggggctttgtga-3' |
| 154-F (321 bp) | 5'-tgggacactgcctgtatgat-3' |
| 118-F (285 bp) | 5'-tgtggctttgccttgtcac-3' |
|  |  |
| B2-R | 5'-atggtcgaggactcagatgt-3’ |
|  |  |
| Amplification of *SPANX-N* in dog |  |
| Dogspa1 Fo/ Dogspa1 Re (710 bp) | 5'-cacccacagagatgtcagaa-3'/5'catcctcatgagacacagga-3' |
| Dogspa2 Fo/ Dogspa2 Re (606 bp) | 5'-gtcaggaaccctgagataga-3'/5'-tggtctctctgtagttggct-3' |
|  |  |
| **RT-PCR expression of *SPANX-N* in dog** |  |
| Dogspa1 eF/ Dogspa1 eR (573 bp) chrX | 5'-atgaccaaagccaaagggtg-3'/5'-cggggtctcttgtttatcct-3' |
| Dogspa 2 eF/ Dogspa 2 eR (271 bp) chr 31 | 5'-gaatgccatgcaccaaagga-3'/5-catgtagataggcccagatc-3' |
