# Supplementary material for: Evolutionary Diversification of SPANX-N Sperm Protein Gene Structure and Expression
Source: PLoS One. 2007 Apr 4;2(4):e359. doi: 10.1371/journal.pone.0000359 (PMC1831492; doi:10.1371/journal.pone.0000359)
Supplement: Table S2 — Accession numbers of SPANX-N sequences (0.05 MB DOC) [file pone.0000359.s007.doc]

**Table S2. Accession numbers of *SPANX-N* sequences**

| **Sequence** | **Accession no.** |
| --- | --- |
| chimpanzeeSPANX-N2_cDNA | DQ336131 |
| chimpanzeeSPANX-N3_cDNA | DQ336132 |
| DogSPANX-N_Var1 | DQ336122 |
| DogSPANX-N_Var2 | DQ336123 |
| DogSPANX-N_Var3 | DQ336124 |
| DogSPANX-N_Var4 | DQ336125 |
| WolfSPANX-N_Var1 | DQ336116 |
| WolfSPANX-N_Var2 | DQ336117 |
|  |  |
| HumanSPANX-N1_Var1 | DQ336118 |
| HumanSPANX-N1_Var2 | DQ336119 |
| HumanSPANX-N1_Var3 | DQ336120 |
| HumanSPANX-N1_Var4 | DQ336121 |
|  |  |
| HumanSPANX-N2_Var1 | DQ336111 |
| HumanSPANX-N2_Var2 | DQ336112 |
| HumanSPANX-N2_Var3 | DQ336113 |
| HumanSPANX-N2_Var4 | DQ336114 |
| HumanSPANX-N2_Var5 | DQ336115 |
|  |  |
| HumanSPANX-N3_Var1 | DQ336128 |
| HumanSPANX-N3_Var2 | DQ336129 |
|  |  |
| HumanSPANX-N4_Var1 | DQ336126 |
| HumanSPANX-N4_Var2 | DQ336127 |
| HumanSPANX-N5_Var1 | AY823033 |
| MouseSPANX | DQ336130 |
