# Supplementary material for: Evolutionary Diversification of SPANX-N Sperm Protein Gene Structure and Expression
Source: PLoS One. 2007 Apr 4;2(4):e359. doi: 10.1371/journal.pone.0000359 (PMC1831492; doi:10.1371/journal.pone.0000359)
Supplement: Table S3 — Primers used for EMSA analysis (0.03 MB DOC) [file pone.0000359.s008.doc]

**Table S3. Primers used for EMSA analysis**

**Oligonucleotide name Sequence (5’-3’)**

_____________________________________________________________________________________

C244F 5’-aaatgtagccacactagcgt-3’

C244R 5’-caaaccaaatgctgttgtt-3’

C170F 5’-gaacattaaacatggagaaatg-3’

C170R 5’-ctagggtggcttcacatca-3’

C209F  5’–aagcccagggagtggtcag-3’

C209R 5’- tggttcttcaatgtctgcgg-3’

N173F 5’–atcacttcataggaatggct-3’

N173R 5’–acagggatgtagaggaaac-3’

N159F 5’–ttgggatgcatcttcagggg-3’

N159R 5’–atacaggcagtgtcccagcc-3’

N229F 5’–gtgcttcgtgatgtcaaag-3’

N229R 5 -gctgttccatgattctggtt-3’

14F 5’-ggtcagcagtggggctttgt-3’

C209R 5’- tggttcttcaatgtctgcgg-3’

31F 5’-gtgatgtgaagccaccctag-3’

C209R 5’- tggttcttcaatgtctgcgg-3’

51F 5’-ggctgccattggctgggaca-3’

C209R 5’- tggttcttcaatgtctgcgg-3’

13R   5’-ctgcggcaggcttttgtagg-3'

C209F 5’–aagcccagggagtggtcag-3’

34R 5’-ttttgaatcttcgcagtggcc-3'

C209F 5’–aagcccagggagtggtcag-3’

52R 5’-tggcccggagctcttgccct-3'

C209F 5’–aagcccagggagtggtcag-3’ ____________________________________________________________________________________
