# Supplementary material for: Evolutionary Diversification of SPANX-N Sperm Protein Gene Structure and Expression
Source: PLoS One. 2007 Apr 4;2(4):e359. doi: 10.1371/journal.pone.0000359 (PMC1831492; doi:10.1371/journal.pone.0000359)
Supplement: Table S4 — Nucleotide and amino acid variants in the canine SPANX-N coding region (0.07 MB DOC) [file pone.0000359.s009.doc]

**Table S4A** Nucleotide and amino acid variants in the canine *SPANX-N* coding region

Gene

Allele Nucleotide AA changes Nucleotide AA changes Nucleotide

Exon1 Exon1 Exon2 Exon2 Intron

*SPANX-N* 1 G/A - A/G; A/G N/S G/A; C/T; A/G

(61)* (316; 404) (232; 235; 250)

2 - - - - -

3 - - A/G; A/G N/S G/A; A/G

(316; 404) (232; 250)

4 - - A/G N/S -

(404)

______________________________________________________________________________________

* In parenthesis - the locations of changes.

In red - the nucleotide changes leading to amino acid substitutions.

**Table S4B. Multibreed plate**

| **Breed** | ***SPANX-N* gene** |
| --- | --- |
| Gordon Setter | variant 1 |
| Catahoula Leopard Dog | variant 1 |
| Spinone Italiano | variant 1 |
| Miniature Bull Terrier | variant 1 |
| Maltese | variant 1 |
| Sussex Spaniel | variant 2 |
| Chihuahua | variant 1 |
| Schnauzer, Miniature | variant 2 |
| Papillon | varinat 1 |
| Pug | variant 1 |
| Staffordshire Bull Terrier | variant 2 |
| Brussels Griffon | variant 1 |
| Schnauzer, Standard | variant 1 |
| Mastiff | variant 1 |
| Otterhound | variant 2 |
| Tibetan Terrier | variant 2 |
| Saint Bernard | variant 1 |
| Boston Terrier | variant 1 |
| Clumber Spaniel | variant 1 |
| Wirehaired Pointing Griffon | variant 4 |
| Fox Terrier, Smooth | variant 1 |
| Scottish Terrier | variant 2 |
| Poodle, Toy | variant 1 |
| Affenpinscher | variant 2 |
| Boykin Spaniel | variant 2 |
| American Staffordshire Terrier | variant 1 |
| Bouvier Des Flandres | variant 2 |
| Great Dane | variant 1 |
| English Springer Spaniel | variant 1 |
| Lakeland Terrier | variant 2 |
| Siberian Husky | variant 1 |
| Brittany | variant 3 |
| Soft Coated Wheaten Terrier | variant 1 |
